# Comparative study of immune responses and intestinal microbiota in the gut-liver axis between wild and farmed pike perch (*Sander Lucioperca*)

**DOI:** 10.3389/fimmu.2024.1473686

**Published:** 2024-10-08

**Authors:** Jing Wang, Shaowu Li, Zhipeng Sun, Cuiyun Lu, Ran Zhao, Tianqi Liu, Di Wang, Xianhu Zheng

**Affiliations:** ^1^ Heilongjiang River Fisheries Research Institute, Chinese Academy of Fishery Sciences, Harbin, China; ^2^ Key Laboratory of Aquatic Animal Diseases and Immune Technology of Heilongjiang Province, Heilongjiang River Fisheries Research Institute, Harbin, China; ^3^ Key Laboratory of Freshwater Aquatic Biotechnology and Breeding, Ministry of Agriculture and Rural Affairs, Harbin, China; ^4^ National and Local Joint Engineering Laboratory of Freshwater Fish Breeding, Heilongjiang River Fisheries Research Institute, Harbin, China

**Keywords:** *Sander lucioperca*, hematological, metabolic enzyme, gene expressions, intestinal flora

## Abstract

**Introduction:**

Pike perch (*Sander Lucioperca*) is a predatory freshwater fish, which is highly popular amongst consumers, owing to its white flesh with a delicate structure and mild flavor. Compared to wild pike perch, the diet of farmed ones has shifted from natural food to artificial feeds. These changes would affect the gut flora of the pike perch. Endogenous metabolites of the intestinal flora are transferred through the gut-liver axis, which affects the physiological functions of the host. By studying wild and farmed individuals of the pike perch, novel insights into the stability of the intestinal flora can be provided.

**Methods and results:**

In this study, we measured various immune parameters in the blood, liver and intestine of wild and farmed pike perch using enzyme activity assays and real-time fluorescence quantitative PCR. Gut microbes were also collected for 16S rRNA gene sequencing. Our results showed that the serum low-density lipoprotein cholesterol (LDL-C) levels were twice as high in the wild group as in the farmed group. Furthermore, the activities of glutamate pyruvate transaminase (GPT) and glutamate oxaloacetate transaminase (GOT) in the intestinal tissues of the wild group were 733.91 U/g and 375.35 U/g, which were significantly higher than those of the farmed group. Expression of *IL10* in the liver of farmed pike perch was also 4-fold higher than that of wild pike perch. The expression of genes related to the p53-BAX/Bcl2 signaling pathway was higher in both intestinal and liver tissues of wild pike perch compared with farmed. 16S rRNA gene analysis of the gut microflora showed a high relative abundance of *Cetobacterium* in the gut of farmed pike perch.

**Conclusion:**

As a result, our study indicates that dietary differences affect the diversity, composition and relative abundance of the gut flora of the pike perch. Meanwhile, it affects the glycolipid metabolism and immunomodulation of pike perch.

## 1 Introduction

Interactions between diet and gut flora play a major role in regulating metabolic functions in vertebrates (95). The gut microbial communities in fish are extremely complex and may differ according to nutritional status, age, and environmental and other factors (1, 2). The lifestyle of fish directly affects the formation of the gut flora, and bacteria specialized to their dietary intake will thrive (3). Gut microbes provide the host with nutrients, extracellular enzymes, fatty acids, and vitamins that cannot be obtained from outside sources (4). Numerous studies have shown that feed formulation can influence gut flora homeostasis and metabolism (5, 6). Therefore, developing a better understanding of the function of bacteria in the fish gut would be beneficial to improving fish farming practices (7, 8). Studying the variations between wild and farmed fish of the same species can help us to better understand the stability of individual gut microbial species (9).

The liver and gut are important sites of immune metabolism in aquatic organisms and the synergistic regulation of the gut-liver axis in fish has been recently recognized (10). The gut-liver axis refers to interactions between the gut, gut flora and liver, and it plays a crucial role in maintaining immune homeostasis (11). Gut mucosal surfaces act as biophysical barriers and secrete mucus to protect against pathogens (12). Additionally, gut-associated lymphoid tissue (GALT) is a crucial component of mucosal immunity, housing a diverse array of immune cell types that facilitate immune surveillance (13). The liver is a central immune organ, adjacent to GALT (14). Liver carries out immune surveillance of pathogens entering through the gut, and it is also affected by changes in the microbiota (15). The endogenous metabolites of gut flora (such as bile acids and amino acids) are transported to the liver via the portal vein, where they can modulate liver functions including enterohepatic exchange (16). Studies have shown that external factors, such as the farming environment and food provided, affect the homeostasis of gut microorganisms and that disturbances in the gut microecology further affect the metabolism and physiological homeostasis of the host (17, 18). However, differences in the mechanisms of gut-liver axis regulation between wild and farmed fish remain unclear.

Hematologic indicators can indicate metabolic disorders and stress and play an important role in analyzing the health status of animals (19). Blood biochemistry can therefore provide valuable insights into an organism’s internal environment (20). The levels of blood or plasma ions and enzymes with significant metabolic roles are established measures for assessing the health of fish (21). Environmental factors including temperature, salinity, oxygen content, and water quality may all affect these indicators (94). Feeding schedules and stocking densities can also have an impact (22). For instance, during hypoxic stress, concentrations of cholesterol, glucose, and other vital blood components are altered considerably ( 23, 24).

When fish are raised in confinement, their environment and habitat structure differ and their diet changes from natural food to artificial feed (4). These changes affect fish growth, immunity, and gut microbiota, all of which play important roles in fish health (25). The pike perch (*Sander lucioperca*) is a carnivorous fish, distributed throughout Eurasia, and it has a high economic value (26). With the advantages of fast growth, high nutritional value and disease resistance, commercial farming of the pike perch is growing (27). In this study, we compared differences in blood biochemistry, key enzyme activities, the expression of immune-related genes and gut flora composition between wild and farmed populations of pike perch. Our objectives were: (1) to characterize the changes in serum biochemistry between wild and farmed pike perch; (2) to compare the differences between the immunity indexes of the two fish populations; and (3) to evaluate the effects of different environmental feeding conditions on the intestinal flora of pike perch. In this way, the adaptations and potential immune mechanisms of pike perch populations under different environmental conditions can be evaluated.

## 2 Materials and methods

### 2.1 Ethics statement

All animal procedures in this study were conducted with the approval of the Animal Care Ethics and Experimentation Committee of Heilongjiang Fisheries Research Institute, Chinese Academy of Fisheries Sciences.

### 2.2 Animal collection and tissue sampling

Five wild pike perch (3-year-old, 1530 ± 170 g) were collected in April 2023 from Khanka Lake, located in the upper reaches of the Wusuli River in Heilongjiang Province (45°37’N; 132°49’E). At the same time, five farmed pike perch (3-year-old, 1260 ± 150 g) were collected from Hulan Experimental Farm at the Heilongjiang Fisheries Research Institute, Chinese Academy of Fisheries Sciences. Farmed pike perch are bred by the Heilongjiang Fisheries Research Institute and is fully adapted to artificial feeding (Dongyu Bio, Zhejiang, China). The main ingredients of the feed include: fishmeal, flour, soya bean meal, fish oil, soya bean lecithin oil, di-calcium phosphate, vitamins and minerals.

Samples were taken as soon as possible after capture. Before sampling, the fish were anesthetized with 150 mg/L MS-222 (Sigma-Aldrich). The serum, liver and intestine of each fish were collected under sterile conditions and stored at -80°C. The digestive tract was rinsed five times with sterile PBS to remove intestinal contents. The mucus was then removed by scraping with a sterile scalpel and collected into a 1.5-ml sterile microcentrifuge tube. DNA was extracted from each mucosal sample for subsequent 16S rRNA gene sequencing.

### 2.3 Hematologic and biochemical analyses

Serum parameters were measured using a hematology analyzer (BS-240 Vet, Mindray). Serum levels of Complement 3 (C3), Complement 4 (C4) and Immunoglobulin M (IgM) were measured with ELISA kits (Jianglai, Shanghai, China), according to the instructions of the manufacturer.

### 2.4 Enzyme activity assays

Tissues (1 g) were homogenized and the supernatant extracted for enzyme activity assays. Glutamate pyruvic transaminase (GPT), glutamate oxaloacetic transaminase (GOT), trypsase (Try), lipase (Lip), amylase (AL), malondialdehyde (MDA), superoxide dismutase (SOD), catalase (CAT), alkaline phosphatase (AKP), acid phosphatase (ACP), and lysozyme (LZM) levels were measured with the procedures provided by Grace Biotechnology, Suzhou, China.

### 2.5 Gene expression analysis

RNA was extracted from the stored tissues with the RNAiso Plus kit (Takara, China). Total RNA was reverse transcribed into cDNA using the PrimeScript™ FAST RT reagent Kit with gDNA Eraser (Takara, China). This was followed by quantitative real-time PCR (qPCR) on an ABI 7500 Real-Time PCR System using the kit TB Green Premix Ex Taq ™ II FAST qPCR (Takara, China). NCBI (https://www.ncbi.nlm.nih.gov/) was utilized to design the required primers (detailed in **Table 1**). Once qPCR was complete, the Ct values for the target gene and *GAPDH* were obtained for each sample, and the 2^−ΔΔCT^ method was used to determine the relative gene expression levels.

**Table 1:** Used primers for target genes of q-PCR in the present study.

Target gene	Primer sequences (5′-3′)	Accession number
*GAPDH*	F: TGCTCACTTGAAGGGTGGTG R: TCAGGCCCTCAATGATGACG	XM_031299603.2
*BAX*	F: CAGGGTGGTTGCACTGTTCT R: GATCGAATACCCTCCCAGCC	XM_031312263.1
*Bcl-2*	F: TTGTCCCTCCACCGAGTTTG R: CGTTTGCAGAGGTGAGGGAT	XM_031294226.2
*P53*	F: ACACGACGGAAAAGGGAGAC R: CTGACACGACTGAACACCGA	XM_031298412.2
*Caspase3*	F: GAGTGGAGCTGGATAACGGAT R: GCATGAACCAGGAGCCATTG	XM_031280131.2
*Caspase9*	F: TTATGGTGTGGACGGACAGC R: GTTCAACCTCGTCAGGGGAC	XM_031286531.2
*Mpx*	F: GCAAACTACAGGGAGGACCC R: CCATGTTGAGAGAGCCCAGG	XM_031278045.2
*Mpeg*	F: TCACCAAAGCCAAAAGCTGC R: CAGCGGGTTGTTTGACTGAC	XM_031309065.2
*Occludin a*	F: AGTCCACCTCCCTACGACTC R: TTTAAGGATGCCTGGCGGAG	XM_035991813.1
*Occludin b*	F: TGAGCCTCATGGGTATGGGA R: AGCCATAGGATCCGCCAATG	XM_031300916.2
*Claudin 12*	F: TTCACCCTTCTGGTTGCCTC R: GCAACCACACTTTGCACGTA	XM_031299250.2
*Claudin 15*	F: AGGAGTGCAGTGTTCGAAGG R: TTGAAGGCGTACCAGGACAC	XM_031289825.2
*HO-1*	F: TTACTTCCCGGCTGAACTGG R: CAGGTTGGGACTGGACACAC	XM_031285861.2
*Keap-1*	F: ATCCCAGGGTTATGGACCGA R: CTGGTACATGACAGCACCGT	XM_031285821.2
*Nrf2*	F: CACAGAAGAGAACGGTGACG R: GTAGTAGTGGACCTGGGGAGA	XM_031297154.2
*SOD*	F: ATAATCACCCTCACTGGCCC R: CCAGCATTGCCCGTCTTTAG	XM_031280860.2
*CAT*	F: ATGTGGCACGCTACAACAGT R: CCTGCCATGTTCTGGCAAAG	XM_031316748.2
*GPX*	F: CTGGTCAATGCTGACGGTGA R: GCATAATCTGGGTTGGTGGC	XM_031301500.2
*IL-10*	F: CCAATGCTGTCGTTTCGTGG R: GTATGCTGTTCATGGCGTGG	XM_031323076.2
*MAPK14*	F: CTGGACACAGACAAGCGGAT R: AATTGTCGGAGGCTGGACTT	XM_031286367.1
*APAF-1*	F: TGTACAGGTGTTGGAGGTGC R: GTCAACAGGCAAGGTGGAGA	XM_031313342.2

### 2.6 Intestinal flora analysis

High-throughput sequencing of the 16S rRNA gene was performed at Personal Biotechnology Co., Ltd (Shanghai, China) using an Illumina MiSeq sequencer (300 bp double-ended reads). Sequencing reads were assigned based on unique barcodes for each sample. Microbiome bioinformatics were performed with QIIME2 2019.4. Briefly, raw sequence data were demultiplexed using the demux plugin following by primers cutting with cutadapt plugin (28). Sequences were then quality filtered, denoised, merged and chimera removed using the DADA2 plugin (29). Non-singleton amplicon sequence variants (ASVs) were aligned with mafft (64) and used to construct a phylogeny with fasttree2 (65). The obtained raw data were analyzed using the QIIME2 cloud analysis system (https://www.genescloud.cn/home).

### 2.7 Data analysis

Data are expressed as means ± standard errors of the mean (SEM). Statistical analyses were performed with SPSS software. Independent T tests were used to compare differences between treatment groups. Significance was determined if *P* < 0.05 and defined as highly significant if *P* < 0.01.

## 3 Results

### 3.1 Hematologic analyses

**Figure 1:**
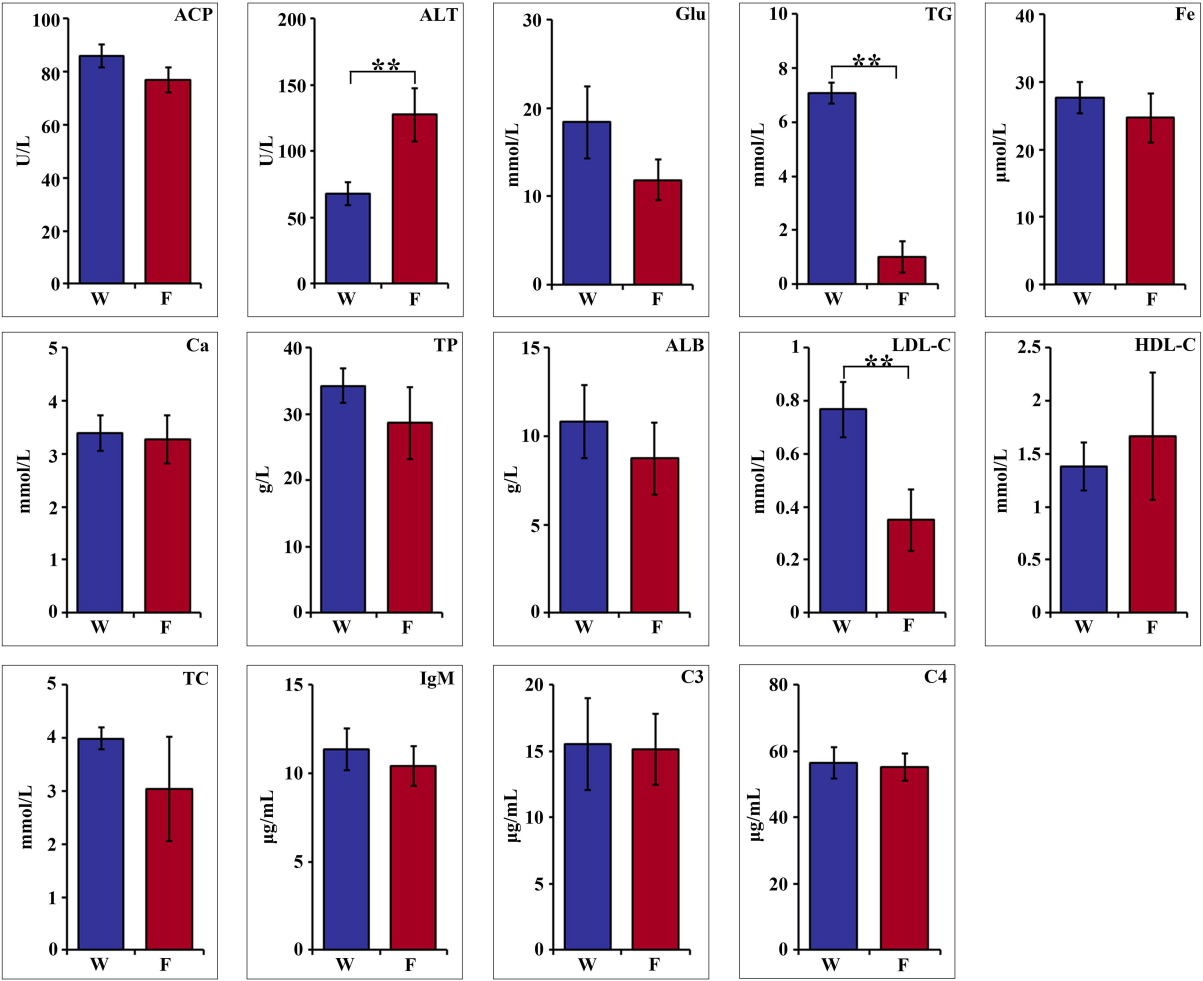
Comparison of hematological parameters of wild (W) and farmed (F) pike perch. Results are shown as mean ± SEM. Asterisks indicate differences between groups W and F. ** highly significant (P < 0.01).

Hematologic parameters are shown in **Figure 1**. Statistical analyses revealed nonsignificant difference in acid phosphatase (ACP), glucose (Glu), Fe, Ca, total protein (TP), albumin (ALB), high-density lipoprotein cholesterol (HDL-C), total cholesterol (TC), IgM, C3 and C4 between the two groups. The serum alanine aminotransferase (ALT) activity of farmed pike perch (F) was 127.47 U/L, which was significantly higher than that of wild pike perch (W) (68.03 U/L) (*P* < 0.01). In contrast, the triglyceride (TG) value in group W was 7.07 mmol/L, significantly higher than that in group F, which was 1.01 mmol/L (*P* < 0.01). Meanwhile, the low-density lipoprotein cholesterol (LDL-C) level in group W was 0.76 mmol/L, which was significantly higher than that in group F, which was 0.35 mmol/L (*P* < 0.01).

### 3.2 Enzyme activity

**Figure 2:**
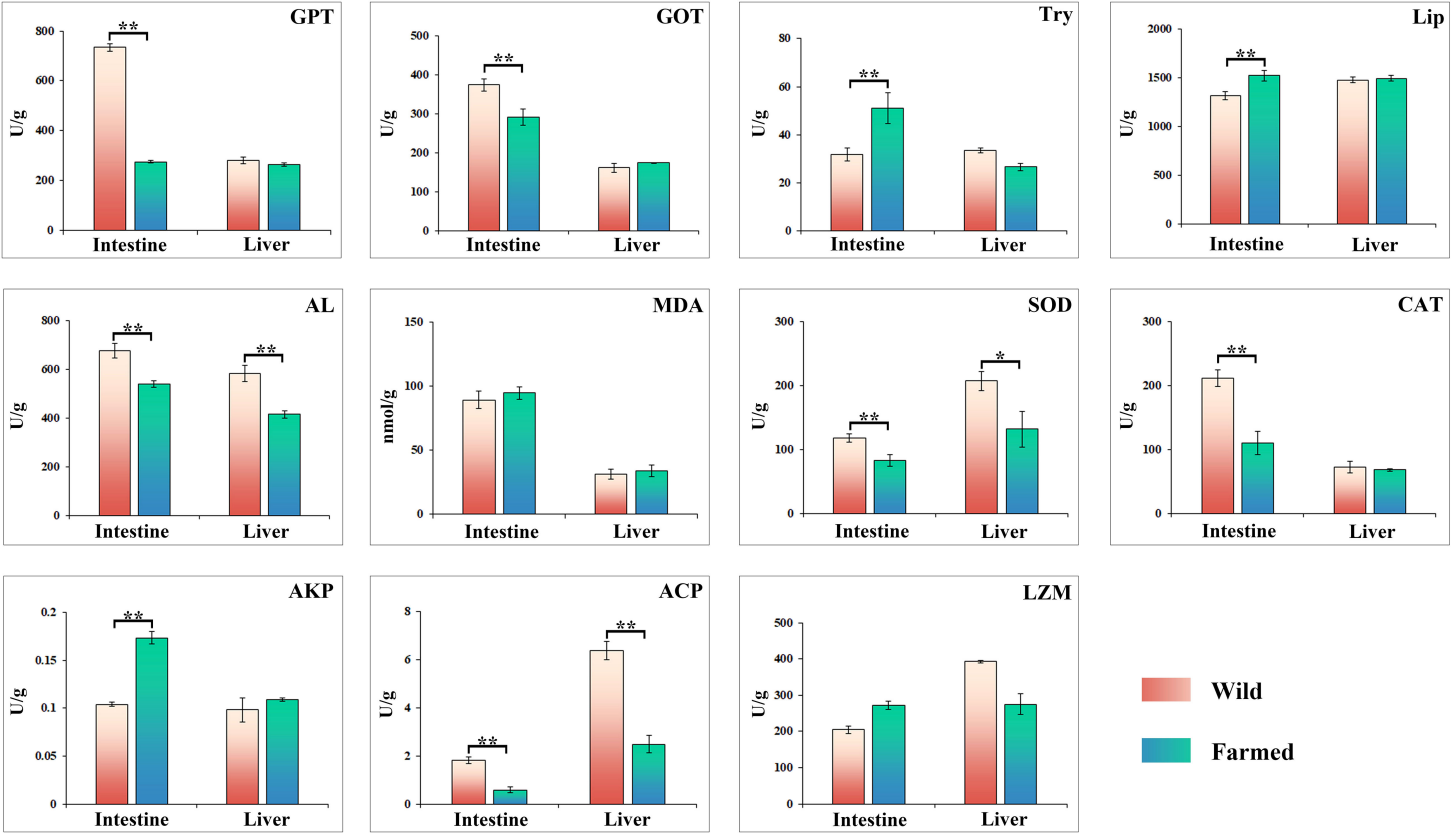
Changes in the activities of digestive and immune related enzymes in liver and intestinal tissues of wild (W) and farmed (F) pike perch. Results are shown as mean ± SEM. Asterisks indicate differences between groups W and F. * Significant (*P* < 0.05), ** highly significant (*P* < 0.01).

The activities of glutamate pyruvate transaminase (GPT) and glutamate oxaloacetate transaminase (GOT) in the intestinal tissues of group W were 733.91 U/g and 375.35 U/g, respectively, which were significantly higher than those of group F (*P* < 0.01; **Figure 2**). Trypsin activity in the intestinal tissue of group F was 51.05 U/g, which was significantly higher than that of group W of 31.75 U/g (*P* < 0.01; **Figure 2**). Moreover, the CAT activity in intestinal tissues of group F was 211.5 U/g, which was significantly higher than that of group W (110.02 U/g). Enzymatic activities of AL, SOD and ACP in both intestinal and liver tissues were significantly higher in group W than in group F (*P* < 0.01; **Figure 2**).

### 3.3 Gene expression analysis

**Figure 3:**
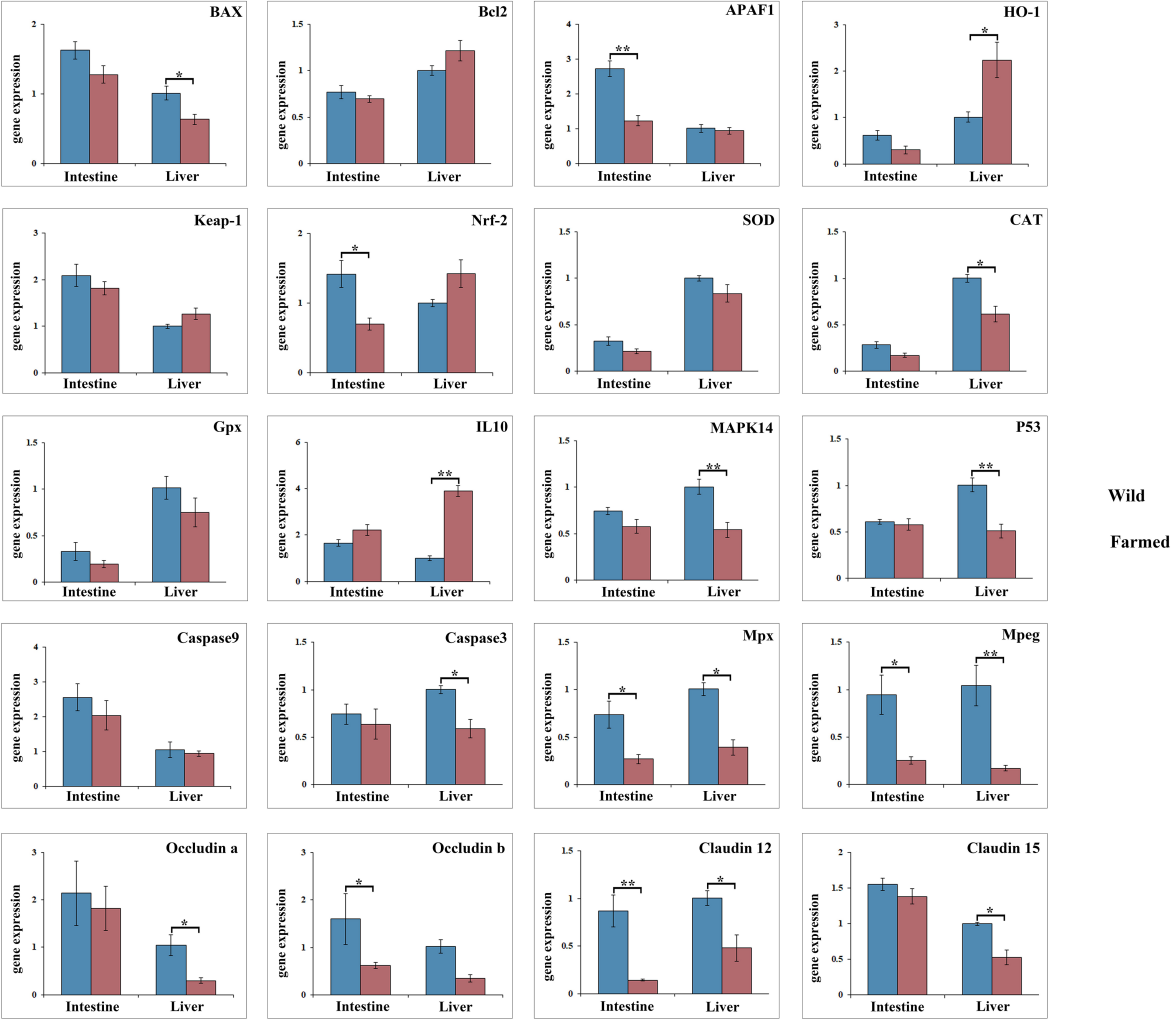
Gene expression analysis of antioxidant genes and anti-inflammatory factors in liver and intestine tissues of wild (W) and farmed (F) pike perch. Results are shown as mean ± SEM. Asterisks indicate differences between groups W and F. * Significant (*P* < 0.05), ** highly significant (*P* < 0.01).

**Figure 4:**
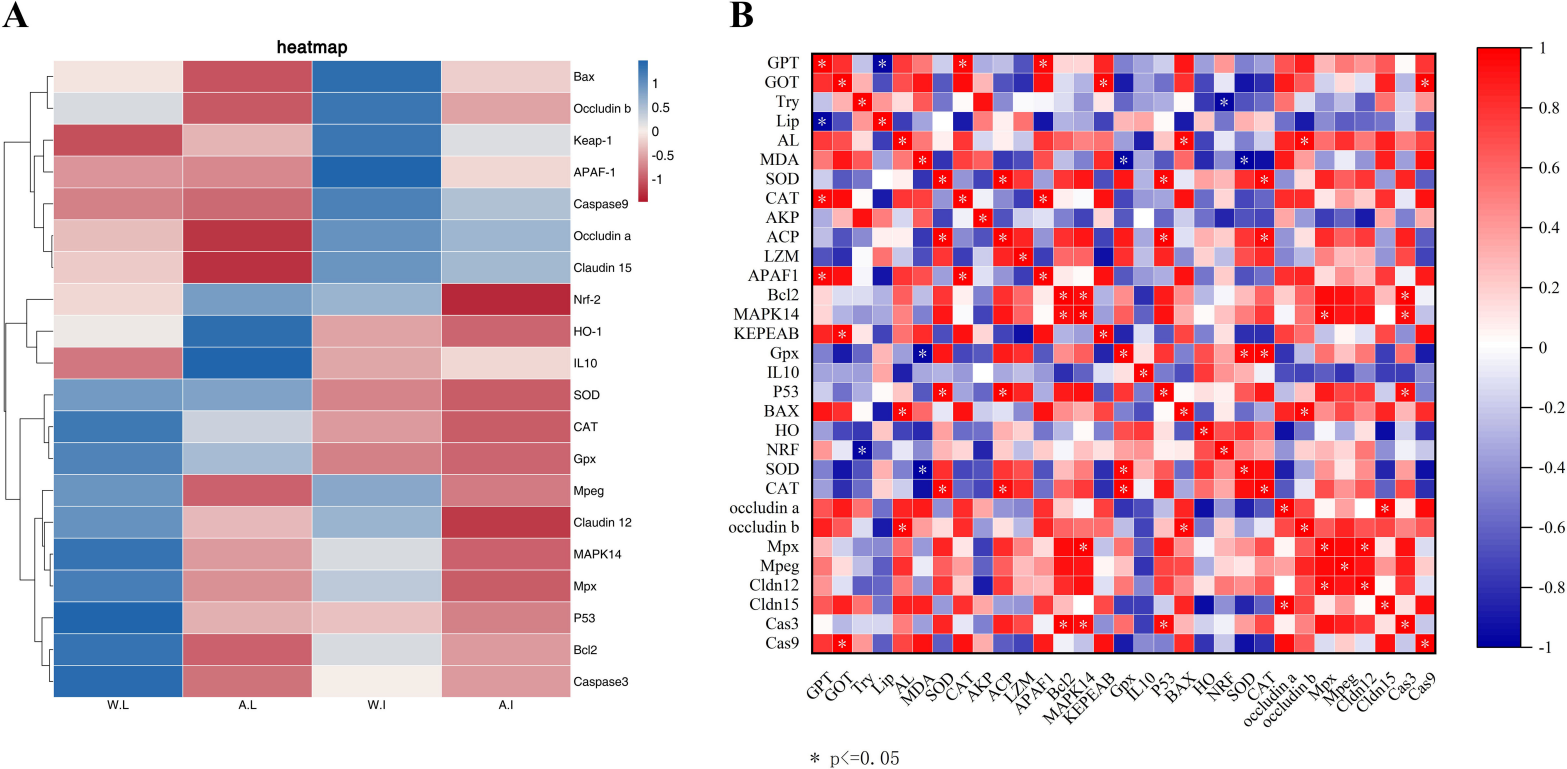
Heatmap of gene expression differences between wild and farmed pike perch in different tissues **(A)**. Red bars indicate higher expression levels and blue bars indicate lower expression levels. Association between 31 markers in gene expression levels and biochemical levels in wild and farmed pike perch **(B)**. Pearson correlation heatmap. Colors range from bright blue (strong positive correlation; r = 1.0) to bright red (strong negative correlation; r = -1.0).

As shown in **Figure 3**, the expression of *Bcl2*, *CAT*, *MAPK14* and *P53* in the liver of wild pike perch was significantly higher than that of farmed fish (*P* < 0.05). Meanwhile, the expression of both *APAF1* and *Nrf-2* in the intestine of group W was significantly higher than that of group F (*P* < 0.05; **Figure 3**). The gene expression of *Mpx*, *Mpeg*, and *Claudn12* in the liver and intestinal tissues of group W was significantly higher than that of group F (*P* < 0.05; **Figure 3**). However, the expression of *HO-1* and *IL10* in the liver of group F was significantly higher than that of group W, and the expression of *IL10* was increased 4-fold (*P* < 0.01; **Figure 3**). As shown in the heatmap (**Figure 4A**), there was a large difference in gene expression in the liver tissues of groups W and F. Pearson correlation analysis showed that GOT enzyme activity was significantly and positively correlated with *Caspase9* expression (**Figure 4B**). While SOD enzyme activity was significantly and positively correlated with *CAT* and *P53* expression (**Figure 4B**).

### 3.4 Comparison of the intestinal microbiome

#### 3.4.1 Differences and diversity in microbiota composition

**Figure 5:**
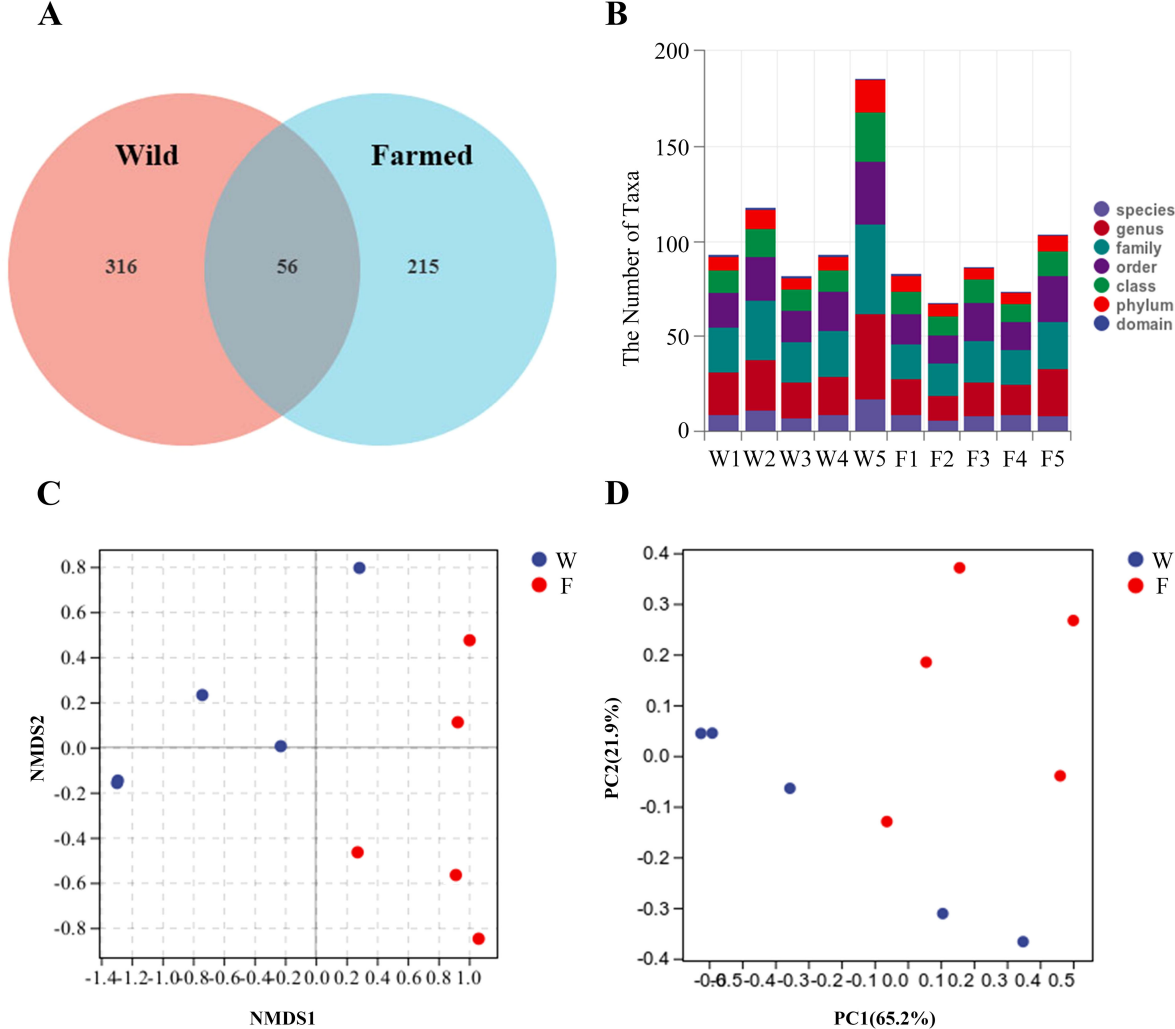
Venn diagram showing the distribution of operational taxonomic units (OTUs) in the gut microbes of wild (W) and farmed (F) pike perch **(A)**. Specific composition of the microbial community in each sample at each taxonomic level **(B)**. Comparison of autochthonous intestine microbiota composition between different groups. Non-metric multidimensional scaling (NMDS) **(C)**, Principal coordinate analysis (PCA) **(D)**.

In total, 587 unique OTUs were obtained from the intestinal contents of the 10-sample fish (**Figure 5A**). A higher number of bacterial taxa can be detected in group W (**Figure 5B**) To allow a more comprehensive assessment of the α-diversity of the microbial communities, richness was characterized using the observed species index Chao1, Simpson’s diversity index, and Faith’s phylogenetic diversity (PD) index, which characterizes evolution-based diversity (**Table 2**).

**Table 2:** The α-diversity indexes of intestinal flora in pike perch.

	Chao1	Faith_pd	Simpson
Wild	104.41 ± 20.96	16.85 ± 3.88	0.3^*^ ± 0.02
Farmed	88.76 ± 7.09	17.38 ± 3.63	0.76 ± 0.01

The β-diversity indicates the effect of the bacterial population on the structure of the bacterial community. Non-metric multidimensional scaling (NMDS) showed that the distance between the W group and F group was large (**Figure 5C**). A principal components analysis (PCA) showed a difference in the bacterial communities of the two fish groups. The samples from the W group clustered more closely together than those of the F group, as shown in the PCA plot (**Figure 5D**).

#### 3.4.2 Changes in microbial communities

**Figure 6:**
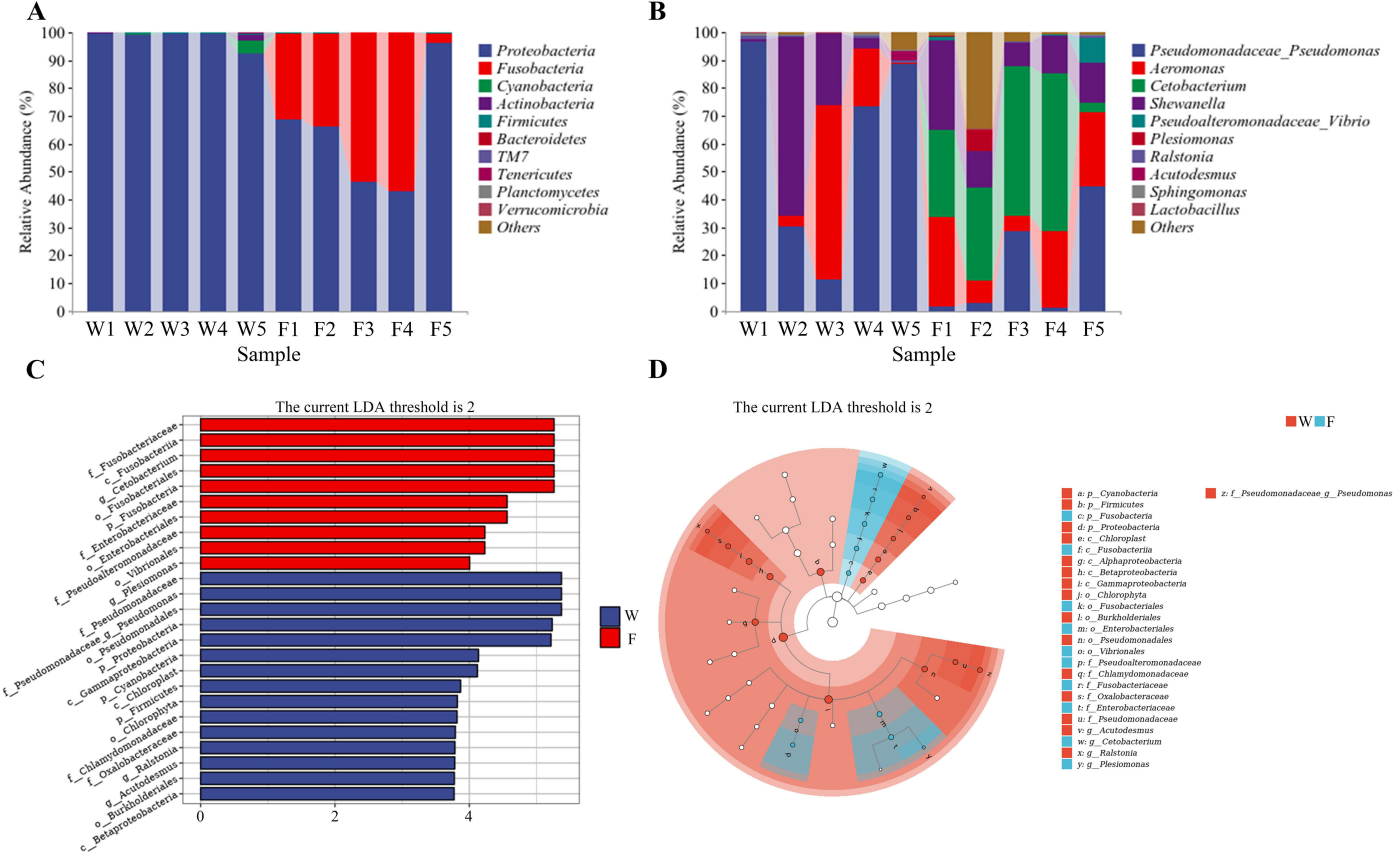
Differences in the relative abundance of the intestine bacteria flora of wild (W) and farmed (F) pike perch. Relative abundance of intestinal bacteria at the phylum level **(A)**; relative abundance of intestinal bacteria at the genus level **(B)**. Linear discriminant analysis (LDA) score of the abundance of taxa **(C)**. LEfSe cladogram **(D)**.

Respective relative abundances of bacteria at the phylum and genus levels are shown in **Figure 6A** and **Figure 6B**. There was no significant difference in the abundances of microorganisms at the phylum level between the two groups. The dominant phylum in group W was Proteobacteria. The dominant phyla in group F were Proteobacteria and Fusobacteria. The diversity of bacterial flora was greater in group F than in group W, and the abundance of *Cetobacterium* was greater in group F (**Figure 6B**).

A linear discriminant analysis (LDA) showed that 25 taxa were significantly enriched, 15 in group W and 10 in group F (**Figure 6C**). According to **Figure 6D**, the abundance of the Firmicutes and Proteobacteria was higher in group W.

#### 3.4.3 Statistical analysis of metabolic pathways based on 16S rRNA gene analysis of intestinal flora

**Figure 7:**
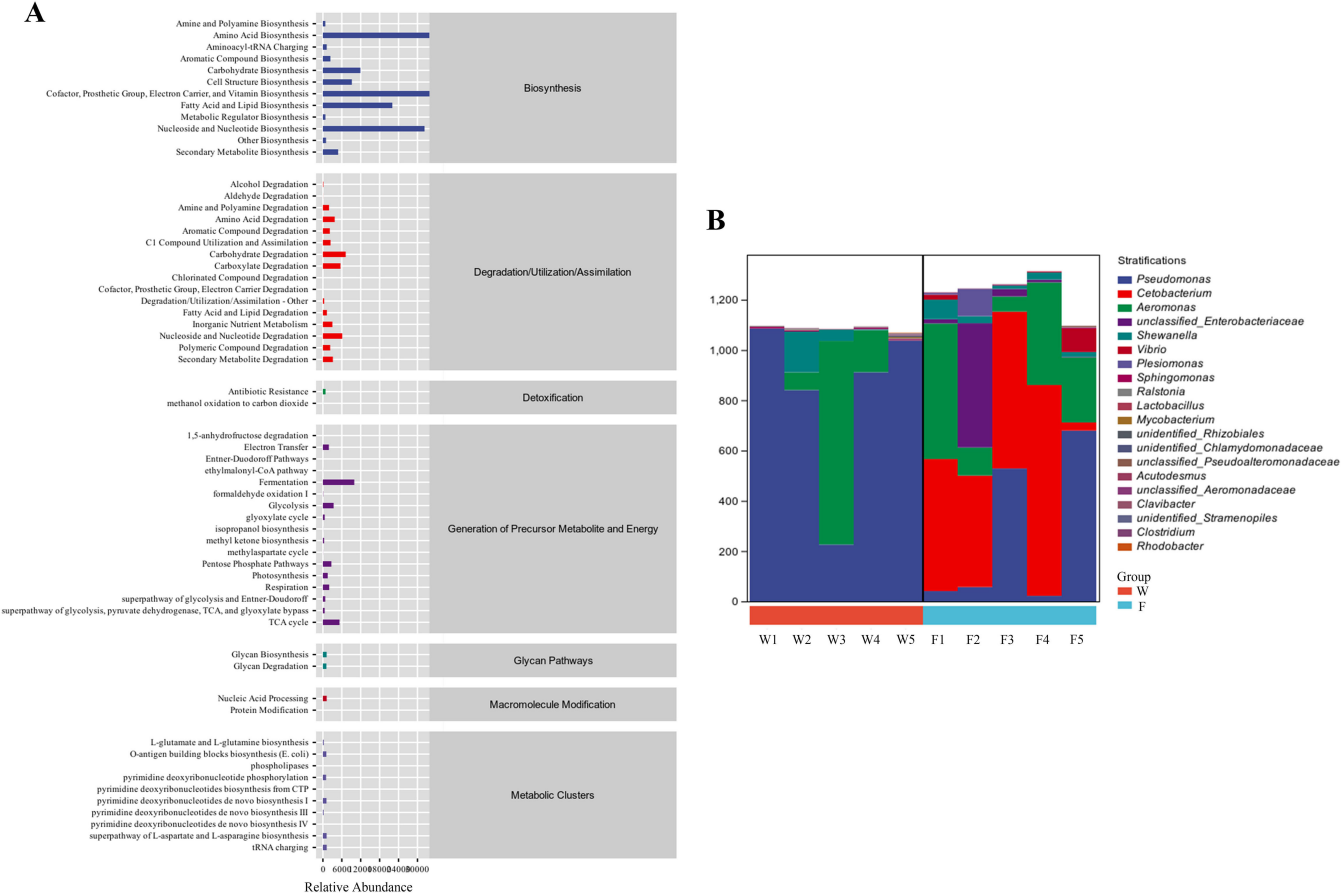
The functions of intestinal microbiota in the fish intestine microbiota had been predicted with the KEGG database **(A)**. Species composition analysis of metabolic pathways **(B)**.

As shown in **Figure 7A**, the function of the intestinal flora in groups W and F included material and energy metabolism and exogenous biodegradation. The species compositions of the metabolic pathways are shown in **Figure 7B**. As shown in **Figure 7B**, the species composition of the two groups consisted mainly of *Pseudomonas* and *Aeromonas* species, and *Cetobacterium* spp. also formed a proportionate part of group F.

## 4 Discussion

This study identifies differences in the gut-liver axis immune response and gut microbiota composition between wild and farmed pike perch. Our physiological, molecular and gut flora results reveal potential immunomodulatory mechanisms by the different intestinal microbial populations of pike perch.

Blood alanine aminotransferase (ALT) and aspartate aminotransferase (AST) play a significant role in amino acid metabolism and liver function in fish (30, 31). In this study, the serum levels of ALT were significantly higher in farmed pike perch than in wild. Similar to our findings, another study has shown that values of ALT in the serum of farmed *Channa argus* are significantly higher than in wild fish (21). This suggests that their metabolic activity is relatively high under farmed conditions, which may lead to elevated concentrations of aminotransferases (32). The levels of total protein, cholesterol and triglycerides can indicate the health status of teleost fish (33), and increased cholesterol levels typically indicate enhanced lipid and lipoprotein metabolism (34). Our results show that the serum levels of TG and LDL-C were higher in the wild group. This suggests that the diet of wild fish is higher in cholesterol or saturated fatty acids compared with the farmed group.

Dietary protein and lipid levels are known to affect the activity of enzymes involved in amino acid catabolism (35). The activities of GPT and GOT have been positively correlated with dietary protein levels and inversely correlated with dietary carbohydrate levels (36). In this study, we found that both GPT and GOT activities in the intestinal tissues of group W were significantly higher than those of group F. This suggests that the wild pike perch has a higher protein content diet. Studies have shown that a high-protein diet increases liver GPT activity in *Eleginops maclovinus* (37 *)*. Antioxidant enzymes also play a crucial role in the innate immune system (38). Studies have shown that when the carnivorous fish *Mylopharyngodon piceus* (39) and *Paralichthys olivaceus* (40) are fed a diet high in carbohydrates, the activity of antioxidant enzymes in their livers is inhibited. In this study, both SOD and CAT enzyme activities were lower in group F than in group W, indicating that the diet of farmed pike perch is higher in carbohydrates.

The gene expression of SOD and CAT demonstrated a similar trend to the enzyme assays, indicating that the high carbohydrate diet reduced the antioxidant capacity of farmed pike perch. Moreover, SOD enzyme activity had a significant positive correlation with *CAT* and *P53* gene expression. *P*53 is a key transcription factor in the regulation of apoptosis, and it induces apoptosis by regulating *BAX* and *Bcl2* gene transcription (41). Bcl-2 was an anti-apoptotic protein that inhibited apoptosis by binding into dimers with the ligand Bax (42). p53 induces apoptosis by upregulating *BAX* and *Caspase3* expression and downregulating the expression of *Bcl2* (41, 43). In this study, the expression of *P53*, *BAX*, *Caspase9* and *Caspase3* in group W was higher than in group F. This suggests that wild pike perch enhance their antioxidant capacity by regulating the expression of apoptosis-related genes in the p53-BAX/Bcl2 signaling pathway.

Interleukin 10 (*IL10*), a pleiotropic regulatory cytokine with anti-inflammatory effects, has been identified in grass carp (44), rainbow trout (*Oncorhynchus mykiss*) (45) and carp (46). Studies have shown that *Taraxacum mongolicum* flavonoids (TMF) can enhance the resistance of the defense system of *Channa argus* by enhancing the expression of anti-inflammatory factor genes (*IL-10*, *tgf-β*) (47). Research in zebrafish has shown that *IL10* can regulate the secretory function of the intestinal mucosa by modulating the number of goblet cells (48). In this study, the expression of *IL10* in the liver of group F was significantly higher than that of group W, indicating that *IL10* plays an important role in regulating the intestinal homeostasis of farmed pike perch. In addition, occludin and claudins have epithelial barrier-forming roles in fish (49, 50). One study showed that the addition of sinomenine to the diet of grass carp resulted in the upregulation of tight junction proteins, which improved intestinal barrier function (51). In this study, these tightly linked genes were all more highly expressed in group W than in group F, possibly indicating that wild pike perch suppress inflammation by controlling epithelial permeability.

The gut microbiota affects the nutrition, development and immunity of the host (52). Diet can act as a very important factor in this context, exerting selective pressure on the microbial composition of the fish gut (53). Typically the gut microbiota produces metabolites that benefit various functions in the host (54). For example, firmicutes facilitate the digestion and absorption of nutrients by breaking down a range of substances (55). Proteobacteria affect host energy accumulation, and an increase in their abundance facilitates the maintenance of energy homeostasis in a hostile environment (56). Studies have shown that *Taraxacum mongolicum* polysaccharide (TMP) fermentation products increased the number of beneficial bacteria in the Jian carp (*Cyprinus carpio* var. Jian) gut, which regulated digestive enzyme activity (57). In this study, the abundance of Proteobacteria in the gut was higher in group W, suggesting that group W accumulates energy more efficiently to cope with a variable environment. This suggests that changes in diet can affect the energy requirements of pike perch. Understanding the composition of the gut microbiota in response to dietary changes would be valuable to establishing practical feeds for pike perch aquaculture.

Gut microbes regulate glucose tolerance and host energy homeostasis via gut microbial metabolites including bile acids, indoles and succinate (58–60). *Cetobacterium* is a prevalent probiotic in the intestinal tract of freshwater fish that produces vitamin B12, which facilitates nutrient absorption (61). Metabolites of *Cetobacterium* have been shown to reduce fat accumulation and enhance the health of the liver and stomach in carp (62). In addition, research in zebrafish has shown that increasing the abundance of acetate-producing *Cetobacterium* alters the gut microbiota to improve glucose homeostasis (63). In this study, the relative abundance of *Cetobacterium* in the gut was higher in group F. In addition, our enzyme activity assays indicated that the diet of group F was higher in carbohydrates. This suggests that carbohydrates in farmed pike perch diets influence the structure of the gut microbiota and *Cetobacterium* enrichment favors glucose utilization. Whether other bacteria play a role in promoting the synergistic regulation of the gut-liver axis in pike perch deserves further investigation.

In conclusion, we showed that wild pike perch diets are higher in protein and cholesterol, whereas the farmed pike perch basal diet was higher in carbohydrates. Dietary differences affect the composition of the gut microflora in pike perch. Metabolites of the gut microbiota further influence glucose metabolism, antioxidant capacity, and immune responses. The gut-liver axis enables pike perch to control and shape the gut microbiota and protect their intestinal barriers. Further studies are required to determine whether pike perch on different diets have specialized gut microbiotas and their effects on their metabolic profile. Additional investigations into the exact metabolic functions of these key gut microbes would also be beneficial to the field.

## Data Availability

The original contributions presented in the study are publicly available. This data can be found in the NCBI repository with the following accession numbers: F1: SAMN43989790; F2: SAMN43989791; F3: SAMN43989792; F4: SAMN43989793; F5: SAMN43989794; W1: SAMN43989795; W2: SAMN43989796; W3: SAMN43989797; W4: SAMN43989798; W5: SAMN43989799.
